# Enriching Earth observation datasets through semantics for climate change applications: The EIFFEL ontology

**DOI:** 10.12688/openreseurope.17992.1

**Published:** 2024-07-02

**Authors:** Benjamin Molina, Carlos E. Palau, Jaime Calvo-Gallego

**Affiliations:** 1 Communication Department, Universitat Politecnica de Valencia, Camí de Vera, s/n, Valencia, 46022, Spain; 2 Computing and Automatics Department, Campus Viriato,scuela Politécnica Superior de Zamora, Avenida de Requejo, 33,, Universidad de Salamanca, Zamora, 49022, Spain

**Keywords:** Ontology and semantics, Climate change mitigation and adaptation, Earth Observation (EO), Essential Climate Variable, Sustainable Development Goals, EO taxonomy

## Abstract

**Background:**

Earth Observation (EO) datasets have become vital for decision support applications, particularly from open satellite portals that provide extensive historical datasets. These datasets can be integrated with in-situ data to power artificial intelligence mechanisms for accurate forecasting and trend analysis. However, researchers and data scientists face challenges in finding appropriate EO datasets due to inconsistent metadata structures and varied keyword descriptions. This misalignment hinders the discoverability and usability of EO data.

**Methods:**

To address this challenge, the EIFFEL ontology (EIFF-O) is proposed. EIFF-O introduces taxonomies and ontologies to provide (i) global classification of EO data and (ii) linkage between different datasets through common concepts. The taxonomies specified by the European Association of Remote Sensing Companies (EARSC) have been formalized and implemented in EIFF-O. Additionally, EIFF-O incorporates:

1. An Essential Climate Variable (ECV) ontology, defined by the Global Climate Observing System (GCOS), is embedded and tailored for Climate Change (CC) applications.
2. The Sustainable Development Goals (SDG) ontology is included to facilitate linking datasets to specific targets.
3. The ontology extends schema.org vocabularies and promotes the use of JavaScript Object Notation for Linked Data (JSON-LD) formats for semantic web integration.

**Results:**

EIFF-O provides a unified framework that enhances the discoverability, usability, and application of EO datasets. The implementation of EIFF-O allows data providers and users to bridge the gap between varied metadata descriptions and structured classification, thereby facilitating better linkage and integration of EO datasets.

**Conclusions:**

The EIFFEL ontology represents a significant advancement in the organization and application of EO datasets. By embedding ECV and SDG ontologies and leveraging semantic web technologies, EIFF-O not only streamlines the data discovery process but also supports diverse applications, particularly in Climate Change monitoring and Sustainable Development Goals achievement. The open-source nature of the ontology and its associated tools promotes rapid adoption among developers

## Plain language summary

Satellites and other tools used to observe Earth provide a lot of data that can help us make decisions, like predicting the weather or understanding climate change. However, these large collections of data are often disorganized and described differently by different people, which makes it hard for scientists and researchers to find and use the information they need.

To solve this problem, the paper introduces the EIFFEL Ontology (EIFF-O). This is a new system designed to organize and link Earth observation data in a way that makes it easier to find and use. It does this by creating common categories and connections between different data sets.

Three important features of EIFF-O are:

It focuses on climate change, helping to categorize important climate data.

It connects the data to global goals for sustainable development, which helps in achieving specific environmental targets.

It links these data sets to the wider internet in a way that makes them easier to share and understand.

The EIFF-O system is freely available for anyone to use and comes with tools that help developers quickly implement it in their projects. This makes it easier for everyone to access and benefit from Earth observation data.

## 1. Introduction

The Earth Observation (EO) domain is expected to grow increasingly in the upcoming years, from €2.8 billion in 2021 to €5.5 billion in 2031 ^1^; not only are satellite services offering vast amounts of real-time and historical data but they are steadily being linked with in-situ data in local areas. Combining local in-situ data with satellite data is difficult because the observed environment differs significantly; however, the expected benefits derived from their synergies in terms of validation and/or accuracy (spatial and temporal) make it worthwhile to promote interoperability and discoverability between them.

Therefore, the management of the related metadata is essential to promote this integration, as well as the rapid development of decision-making applications, which base their forecasts and recommendations on the information provided by local and satellite datasets. In addition to short-term weather forecasts, the climate change domain for adaptation and mitigation policies is most probably one of the top environments under study, as there is a need to consider global and historical data, even if policies are taken only locally (think global, act local).

Data can be global and local but private or open. This study focuses only on open data and publicly available portals to access the EO datasets. Private data are not considered, but two comments are worth mentioning. First, many local decision-makers using open satellite data already own or have access to some sort of (private) local sensor network; thus, they perform integration internally in their own way. Second, some large private data providers are noticing the huge storage capacity required for historical data; considering the fact that most of their revenues come from “fresh” captured data, they are slowly releasing old (historical) data to be stored in larger open systems, thereby contributing to the open community.

The Global Earth Observation System of Systems (GEOSS) ^2^, managed by the Group on Earth Observation (GEO), is probably the best example, as it is a meta-system at a global scale integrating multiple regional, national, and international systems, including both public and private sectors. At the European level, the European Union created the Copernicus programme ^3^ to provide free and open information to service providers, public authorities, and EU citizens. Its implementation is supported by large companies (e.g., ESA, EUMETSAT, ECMWF, and Mercator Ocean).

Unfortunately, the current status of GEOSS does not provide semantic support, searches are mainly keyword-based, and the filters are independent of each other. In addition to geospatial and temporal filtering, only the thematic areas of Climate and Essential Climate Variables (ECVs) seem to be especially relevant for building a search ontology for climate applications.

It is also relevant to mention that ontology usage had been on GEO’s agenda in the past (2009), and an EO committee started to work on highlighting semantic versus semantic interoperability and studying various taxonomies and thesauri, but none of them focused on EO applications at that time ^4^. Sadly, the work was discontinued, and no output ontology was provided or included in the system.

The architecture of GEOSS has experienced significant changes during the last decade, and some categories have been added; however, no clear taxonomy could be found. Searches are still syntactic and use various protocols. OpenSearch ^5^ is the most common interface used by the Data Access Broker (DAB), through which most data providers integrate with GEOSS. Although there are some guidelines for this broker integration process, there is no Linked Data approach, where EO datasets can be semantically published and easily harvested by search engines.

In contrast, Copernicus provides services in six main areas: Copernicus Atmosphere Monitoring Service (CAMS), Copernicus Marine Environment Monitoring Service (CMEMS), Copernicus Land Monitoring Service (CLMS), Copernicus Climate Change Service (C3S), Copernicus Emergency Management Service (CEMS), and security (e.g., border surveillance). As this study focuses on Climate Change, C3S is the most important service under study. Among the different aspects of C3S, and from the perspective of building an ontology, the Common Data Model (CDM) ^6^ is probably the closest approach; it provides a homogeneous format for all data and products in the Climate Data Store (CDS) so that they can be properly processed in the associated toolkit. The CDM is based on the Climate and Forecast (CF) ^7^ convention, but it is mainly restricted to internal toolkit usage. To the authors knowledge, it is not ported or adapted to other platforms, systems, or environments.

In summary, large open EO portals, such as GEOSS and Copernicus, have very limited semantic support and only offer traditional keyword-based (syntactic) search functionality. A huge gap in terms of usage of ontologies and semantics was identified and, therefore, an ontology (EIFF-O) has been developed in order to outperform the current limitations by providing semantic support. More extended information is provided about current related projects and ontologies and their gaps in 8.

EIFF-O is not conceived as a monolithic ontology but as a set of different ontologies to support the different domains covered by the large EO datasets. These domains are potentially large, but we have established three main pillars (general, specific, and goal-oriented):

First, a general approach for classifying a large number of EO datasets is required. This taxonomy has been performed by the European Association of Remote Sensing Companies (EARSC) over the last ten years, with several updates. The latest version ^9^ was specified under the CopHub.AC H2020 project and provided a two-sided view of EO services: (i) a user view, organized in markets and sectors, and (ii) a provider view, split into domains and areas. The specification was never implemented as a formal file (e.g., ontology) to be used by software systems, which represented a clear task to be performed and is presented in this paper.

Second, a specific domain to narrow down the scope and validate the ontology is required. Climate change applications for adaptation and mitigation measures were selected as the target domain, and five pilots were developed under the umbrella of the H2020 EIFFEL project ^10^ supporting all this work. In addition to CF conventions, Essential Climate Variables (ECVs) have been identified as a nuclear set of concepts to work with in Climate Change (CC) applications; therefore, an ontology is required here. ECV taxonomy ^11^ is managed and specified by the Global Climate Observing System (GCOS). Similar to EO taxonomy, there is no formal implementation of ECV taxonomy, another task that was identified and undertaken by the authors.

Third, the discovery of EO datasets with the help of EIFF-O is mostly performed by (data) scientists and developers to build decision-support applications and enhance current policies. Therefore, it seems sensible to tag or link datasets to specific goals. These goals may vary from place to place, but on a global scale, it seems appropriate to start with the Sustainable Development Goals (SDGs) specified by the United Nations. Fortunately, an implemented ontology is available ^12^, including basic concepts such as goals, targets, indicators, and series. This taxonomy has been integrated into the EIFF-O.

Additionally, EIFF-O also promotes the adoption of open vocabularies and formats, such as JavaScript Object Notation for Linked Data (JSON-LD), to shift existing EO datasets into the semantic web and serve as a driver for fast publication and dissemination. Note that having a vocabulary of concepts to tag datasets is essential to produce discoverability and reach the FAIR (Findable, Accessible, Interoperable, and Reusable) principles, but not sufficient to achieve widespread usage. *Schema.org* is a joint initiative (Google, Microsoft, Yahoo) to provide shared vocabularies supporting the embedding of structured data in webpages. A full analysis of the semantics of *Schema.org* is beyond the scope of this section, but there is plenty of related literature ^13^. Essentially, by reusing and extending common concepts (e.g., datasets), EO data can automatically be indexed by global search engines; Google already has a dataset search repository for providers publishing semantically (e.g., JSON-LD) on the web.

Another feature that has attracted widespread use is modularity and extensibility. By default, ontologies can easily be extended by incorporating or linking concepts from other ontologies. In this sense, EIFF-O defines the high-level concept of *Essential Variable*, that is, the ECVs a sub-concept. This allows the introduction of other Essential Variables (EVs) that are defined in other research domains but directly related to EO datasets, such as Essential Agriculture Variables (EAVs) ^14^ and Essential Urban Variables (EUVs) ^15^.

Finally, the work done is not only restricted to the definition and implementation of the ontology, but also includes a set of tools and APIs for easy adaptation and integration with other external components. It is currently being integrated with a Natural Language Processing (NLP) engine to increase the user experience.

The remainder of this paper is structured as follows: Section 2 presents the general methodology used to implement the different ontologies. Section 3 describes the EIFF-O ^16^ ontology and provides a short overview of the four main building blocks. Next, supporting tools were shown to promote and support the use of the EIFF-O. Finally, the paper ends with conclusions and further work.

## 2. Methods

**Figure 1.**
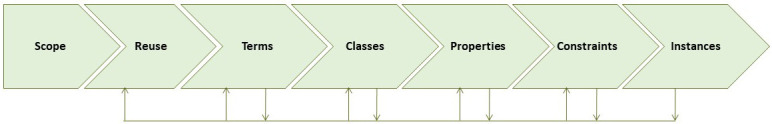
General methodology to build the ontology. This methodology is a didactic and commonly used way to explain the different steps required to build an ontology.

There are various possible and valid ways to follow the ontology-development process. Frequently, the target application itself is the fundamental driver in the analysis and implementation of the ontology, and all the required steps are closely related to it. However, from a general and didactic perspective, we can consider a seven-phase methodology, which is shown in Figure 1 and is based on 17.

The depicted process follows a clear incremental path but includes multiple backward loops if needed. The need for such an action is triggered by evaluating the current step and noticing that something was missing to satisfy the scope of the ontology that should have been defined in a previous step. This assessment, even if strongly influenced by the target application or domain, is also subjective and opens the way to creativity; therefore, multiple valid ways to model a particular domain are valid.

**The first step determines the scope** This phase clarifies the domain under study, as well as the type of knowledge to be managed. Typically, the following questions should be answered: (i) What is the domain? (ii) What is the purpose of the ontology? (iii) What are the types of questions following the competency questions ^18^ recommendation and (iv) who is the target user of the ontology?

**The second step should consider reusing any existing ontologies.** The reuse of existing ontologies (if any) is crucial. One may reuse part (or all) of the available knowledge in one’s domain, thereby reducing the space of uncertainties while connecting things (concepts, entities). Building a new ontology makes sense only if there is no suitable ontology or if the adaptation is too complex. EIFF-O can be considered as a mixture of new concepts (coming from the specification of the different taxonomies) with concepts already available from existing ontologies (e.g., concepts from schema.org, ‘EssentialVariable’ concept from other ontologies, etc.).

**The third step relates to building a terminology.** This phase can be considered a brainstorming process to identify and list the main concepts before starting the later formal steps. Essentially, one should end up with a collection of vocabulary terms stating: (i) the most important concepts to be modelled, (ii) the main properties of these concepts, and (iii) what the target user would say about these concepts. In our case, the main concepts could be easily selected from the three subontologies (EO, ECV, and SDG), and more effort was made to bring the three together under the EIFF-O wrapper and link with schema.org terminology.

**The fourth step defines the classes and the class hierarchy.** This is the formal definition step and is tightly intertwined with the next step, as they are the most critical in the design process. Classes are defined by the ontology designer in a rigorous, open, and creative manner. Furthermore, it also opens the way in which the class hierarchy is built: (i) *Top-down*: from the most general class to the most specialized one, (ii) *Bottom-up*: from the most specific classes (leaves of the tree) to the most general ones; (iii) *Middle-out*: an intermediate approach that allows starting with the clearest and most important concepts and then building the hierarchy by generalizing and specializing additional concepts. The bottom-up approach was used for the EO and ECV ontologies. A top-down approach was employed for the EIFF–O wrapper.

There can be various simultaneous hierarchies as long as they make conceptual sense, and semantic languages do not pose any restrictions and allow reasoning in such scenarios.

**The fifth step defines the properties for each class.** These properties can be data or object properties. Some of them should be available from step 3, and the remaining properties must be accommodated in each class to fulfil the scope. The properties can be *intrinsic* (specific to that class), *extrinsic* (general to other classes), or *relationships* to other classes. After this step, most (if not all) of the competency questions created in step 1 should have been targeted.

## 3. Overview of EIFF-O ^16^

The EIFFEL ontology has been implemented in a modular manner and is composed of four components: (i) the EO taxonomy, specified by EARSC, providing the general-view categorization of EO services; (ii) the ECV taxonomy, specified by GCOS, providing the specific-view categorization for climate change applications; (iii) the SDG taxonomy, specified by the United Nations (UN), providing a goal-oriented perspective; and (iv) the EIFF-O wrapper, establishing the links among the three previous taxonomies and supporting schema.org’s vocabularies for a Linked Data approach. Every module is described separately in the following sections.

Note: Classes are represented by green boxes in the following Figures. These green boxes include the name of the class and number of instances (in brackets). Blue arrows represent ‘rdfs:subclassOf’, which denotes the relationship with SKOS, a common data model for building taxonomies. Green arrows denote the domain-range relationship. Brown boxes denote data properties for the nearby class (green boxes). Pink boxes denote a potential relationship with other subontologies through a nearby class (green box). 

### 3.1. EO ontology

The EO taxonomy has been specified in 9, and it covers two perspectives (market/user vs. thematic/provider), which converge on the concept of EO services.

**Figure 2.**
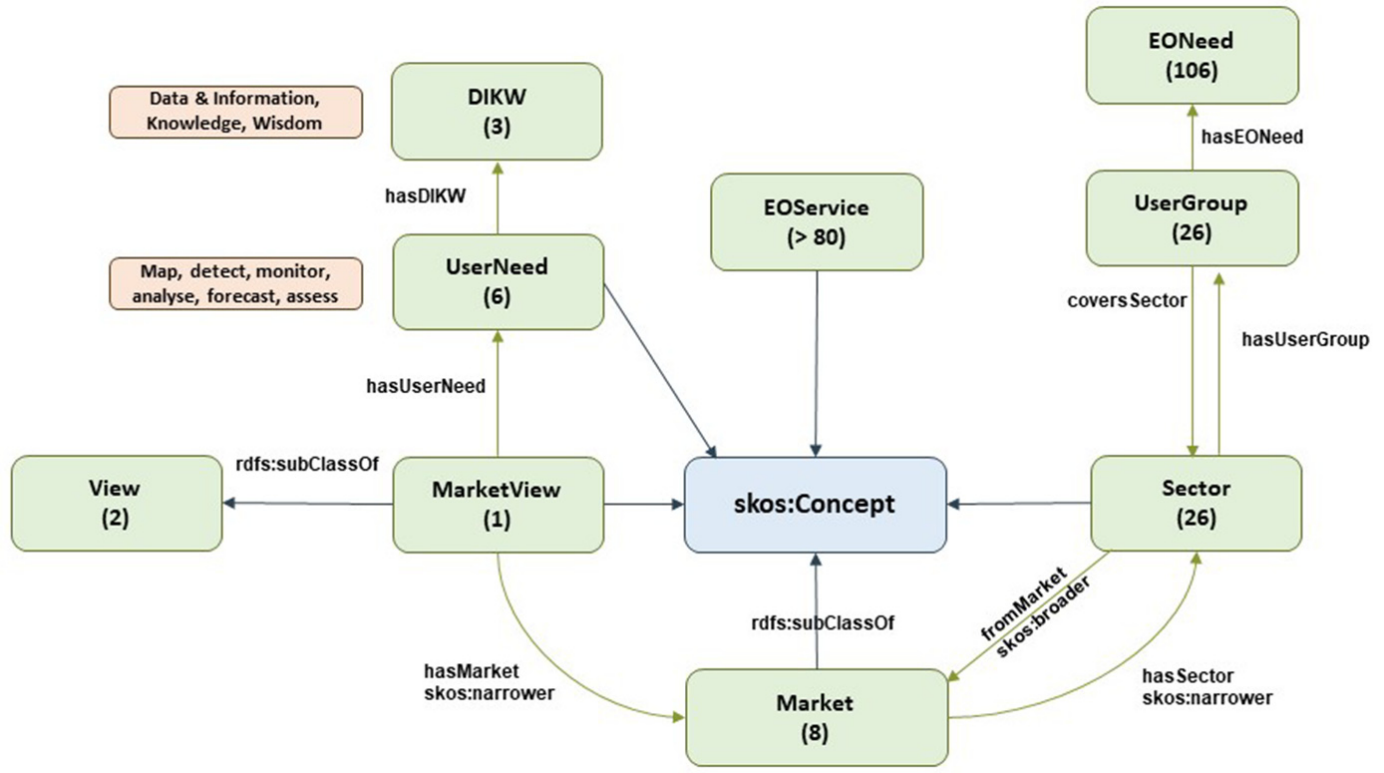
EO taxonomy - market view. EO taxonomy has two main views; the market view (consumer view) shows the perspective of the end-user, and how data consumers search for datasets in their own way. There are 26 different sectors which are grouped in 8 markets.

The market view is shown in Figure 2. The basic overall concept is *View*. A View is a general way of classifying or analyzing EO services; currently, the EO taxonomy only defines two common ways: market and provider views. *MarketView* supports the market-view perspective, as defined by EARSC. *MarketView* is defined by a set of covered *Markets*. The EARSC defines eight different markets (citizens and society, defense and security, urban development, environment and climate, financial and digital services, infrastructure and transport, managed living resources, and energy and mineral resources) in its latest version. According to the diagram in Figure 2, every new version will simply require instantiating a new individual of the *MarketView* class (and adding new *Markets*, if any).

Additionally, *MarketView* is defined by the different operations or needs that the user might have. This is represented by the *UserNeed* class. EARSC defines six main needs (map, detect, monitor, analyze, forecast, and assess) that are mapped to Data & Information, Knowledge, and Wisdom (DIKW) categories. Both are ways of defining the user need and the associated added value (e.g., a “forecast need” will provide more added value to the user than a “map need,” and will require a more complex EO service).

Each *Market* is composed of one or more sectors, represented by the *Sector* class. This narrows down the scope and better defines the target segment. The latest version of the EARSC defines 26 sectors.

Each *Sector* defines a set of potential users or stakeholders interested in consuming EO services related to this specific sector. Such a user profile is represented by the *UserGroup* class, and there is a one-to-one mapping between *Sector* and *UserGroup* according to EARSC. This *UserGroup* class is characterized by one or more EO needs ( *EONeed* class), which are generic expectations from users within a given sector in terms of exploiting EO data.

Finally, the concept of EO services ( *EOService* class) is also included in this market view, but it is better explained by EARSC from the thematic (provider) perspective.

**Figure 3.**
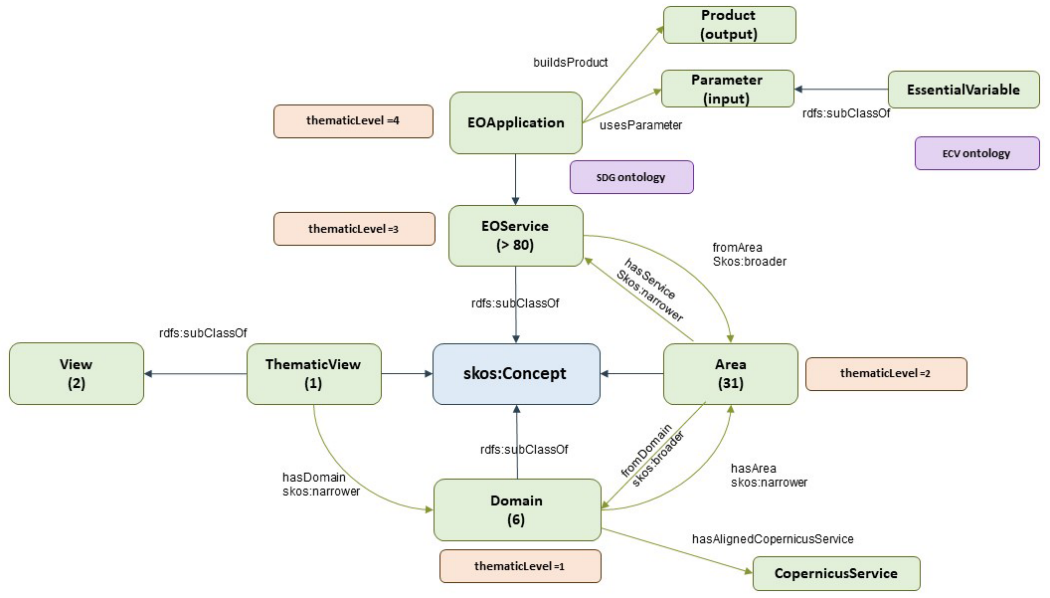
EO taxonomy - provider view. The EO taxonomy has two main views; the provider view shows the perspective of the data provider, and how they categorize their datasets. There are 31 areas grouped in 6 domains.

The provider view is shown in Figure 3. It follows a structure similar to that of the previous market view:

- The *ThematicView* class supports the thematic perspective as defined by EARSC.
- *ThematicView* can be broken down into several *Domains*. This is the first level of segmentation, and EARSC defines six domains (land, built environment, marine and maritime, security and safety, atmosphere and climate, and disasters and geohazards).
- *Domains* are decomposed into *Areas*, which is the second level of segmentation, and EARSC defines 31 different areas. This is equivalent to MarketView’s sector class in the *MarketView*.

The EOService class represents a third level of segmentation according to EARSC, and is a high-level and general representation of a service using EO data. Currently, EARSC defines more than 80 services in different areas.

The thematic view elaborates on the concept of service by introducing a fourth segmentation layer called the EO application (EOApplication class). This is supposed to be a particular implementation of EO services. EARSC does not define any EO applications; however, any implementation available in the market is supposed to fall under this category.

As an EO application, which is a specific implementation of an EO service, it is further composed of a product (product class) and a set of parameters (parameter class). The concept of a product is not clearly defined by EARSC, but points in the direction of a common product offer. The parameters relate to the inputs used by the application to generate the result; one such input is the essential variable (EVs). These variables are critical for observing and monitoring different aspects of Earth’s system, including oceanography, climatology, biodiversity, and geodiversity.

Figure 3 also depicts the links between the given concepts of the EO taxonomy and SDG and ECV ontologies (described in the next sections). ECVs can be included directly as a subclass of EVs. The SDG ontology, through a goal or target concept, can be linked as an additional property of an EO service or EO application.

### 3.2. ECV ontology

**Figure 4.**
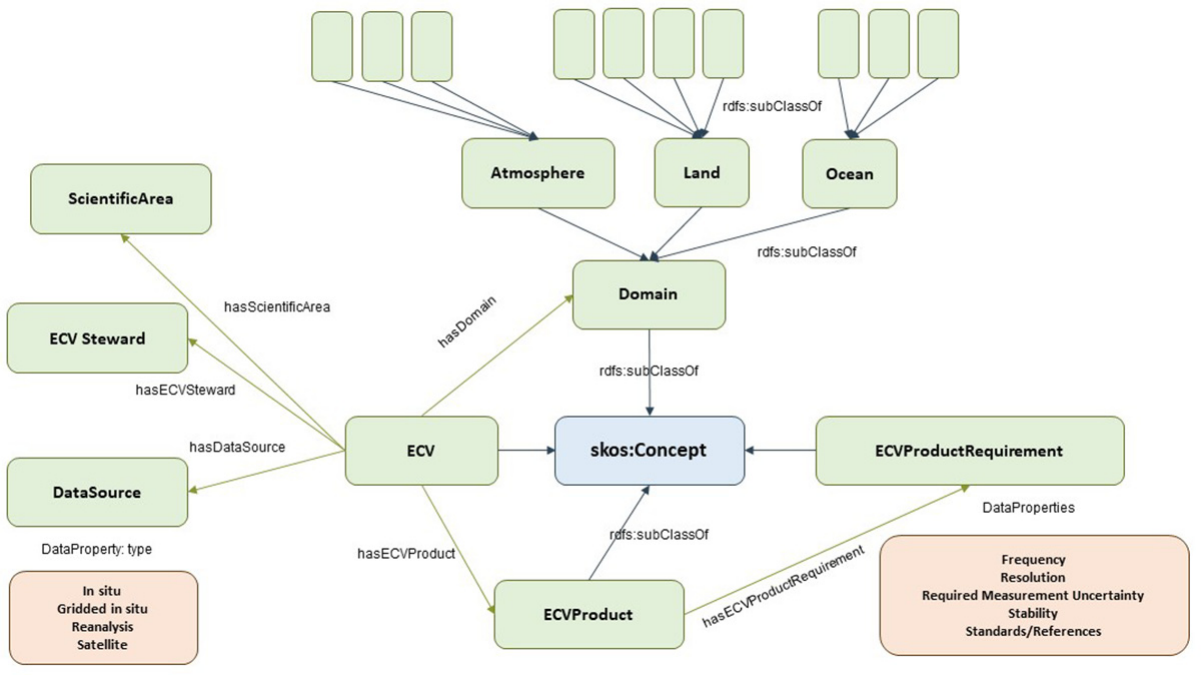
ECV ontology. The Essential Climate Variables (ECV) Ontology is a structured framework that categorizes and defines variables crucial for monitoring the Earth's climate system. Developed to support consistent and comprehensive climate data analysis, the ECV Ontology encompasses variables across atmospheric, oceanic, and terrestrial domains.

The ECV ontology mainly follows the taxonomy guidelines defined by the GCOS, as depicted in Figure 4. This is a good example of building the class hierarchy in a *middle-out* approach by starting with the Essential Climate Variables ( *ECV* class).

ECVs are associated with a *Domain*, which can be *AtmosphereDomain*, *LandDomain* and *OceanDomain*, and are defined as first-level domains. For simplicity, we will omit the ending [-Domain] for these entities (e.g., the OceanDomain class has a rdfs:label annotation with (string literal) value “Ocean”) Each domain includes second-level domains. The *Atmosphere* first-level atmospheric domain includes *Surface*, *Upper-Air* and *Atmospheric Composition* second-level domains. The *Land* first-level domain is composed of *Hydrosphere*, *Cryosphere*, *Biosphere* and *Anthroposphere* second-level domains. The *Ocean* first-level domain includes *Physical*, *Biogeochemical* and *Biological/ecosystems* second-level domains.

The GCOS specifies 54 ECVs split among these 10 second-level domains by 2022. These ECVs were also assigned to six scientific areas (Biosphere, Carbon cycle and other GHGs, Energy and Temperature Hydrosphere, Physical Properties, and Snow and ice).

ECVs had one or more ECV Steward assigned to Steward. They are responsible for the ECVs and manage all related information for a given ECVs. An ECV steward can be assigned to more than one ECV (typically, if they fall under the same domain or scientific area). Currently, there are 31 ECV steps involved.

ECVs have a series of associated data sources that provide information on such ECV. The data sources are classified into four types: in-situ data, gridded in-situ data, reanalysis data, and satellite data. In addition to this category, a data source has a name (typically the name of the provider) and a weblink to access the datasets. The latest specifications include 158 data sources that have been instantiated with the ontology.

ECVs have one or more associated products ( *ECVProduct* class), each with its name, description, and set of requirements. A requirement ( *ECVProductRequirement* class) unit covers various variables such as frequency, resolution, required measurement uncertainty, stability, and related standards/references. Currently, there are 143 *ECVProduct* individuals and 130 *ECVPRoductRequirement* instances.

While instantiating the 54 ECV individuals in the ECV knowledge base, links to the official icon and PDF factsheet (provided on the official GCOS website) have also been included as part of the properties of each ECV.

### 3.3. SDG taxonomy

The United Nations (UN) provides a platform for linked data services ^12^ that is hosted by the Dag Hammarskjöld Library. Currently, it hosts only two resources: (i) the UNBIS Thesaurus and (ii) the Sustainable Development Goals. The ontology associated with these SDGs is hosted in a public GitHub repository ^19^. The approach followed in this ontology is similar to that used in EIFF-O in the sense that it promotes the use of schema.org’s dataset vocabularies and JSON-LD formats to tag the information with metadata, thereby enriching the discoverability.

**Figure 5.**
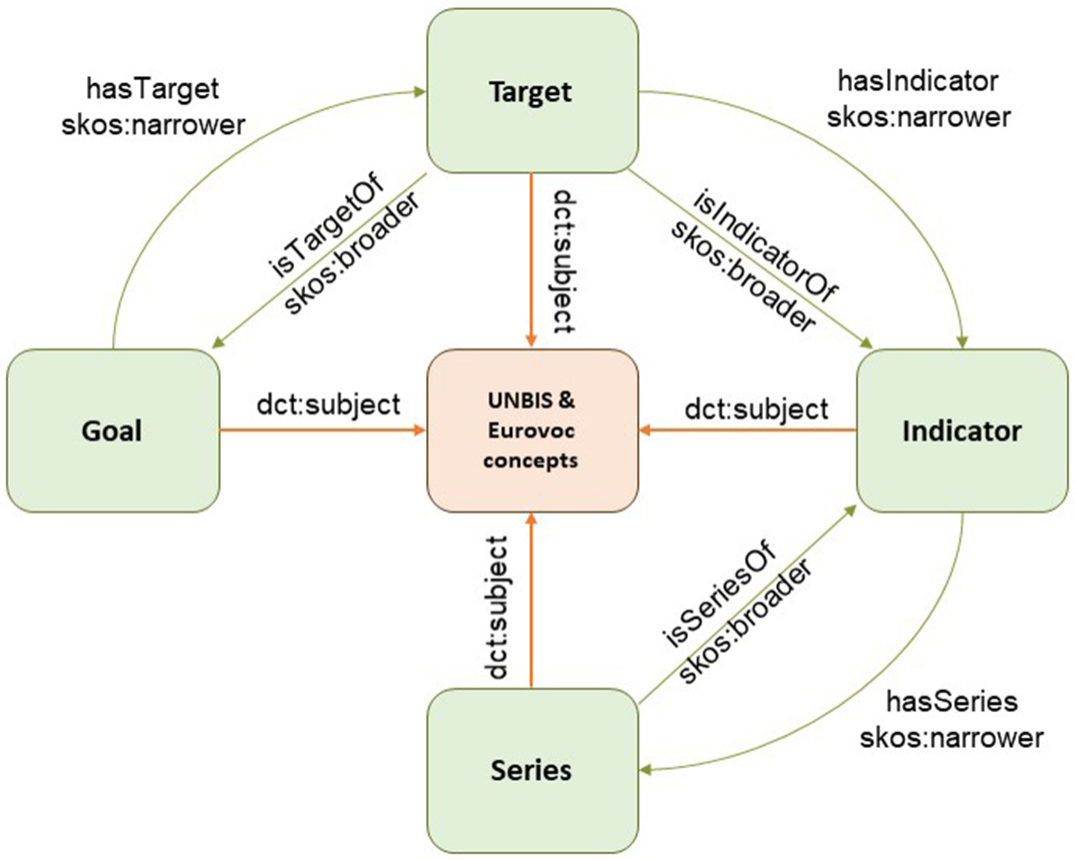
SDG ontology. Source: 18. The Sustainable Development Goals (SDG) Ontology provides a structured framework for representing and linking information related to the United Nations' 17 Sustainable Development Goals. It organizes and categorizes concepts, indicators, targets, and relationships associated with each SDG, enabling clear understanding and integration of diverse data sources.

The SDG ontology is the core part and implements the structure of the SDG goal–target–indicator–series hierarchy, as depicted in Figure 5. The ontology itself is rather simple and uses the SKOS core vocabulary, which is a widely used W3C standard employed by taxonomies and thesauri. Consequently, EIFF-O also uses the SKOS. The SDG knowledge base was provided by instantiating all 17 goals, 169 targets, 232 indicators, and 394 series. property) to external vocabularies, such as UNBIS and Eurovoc, as well as matches with the SDG Interface Ontology (SDGIO) and SDG goals available in Wikidata.

The SDG also includes mappings (via skos:exactMatch property) to external vocabularies, such as UNBIS and Eurovoc, as well as matches with the SDG Interface Ontology (SDGIO) and SDG goals available in Wikidata.

### 3.4. EIFF-O wrapper

**Figure 6.**
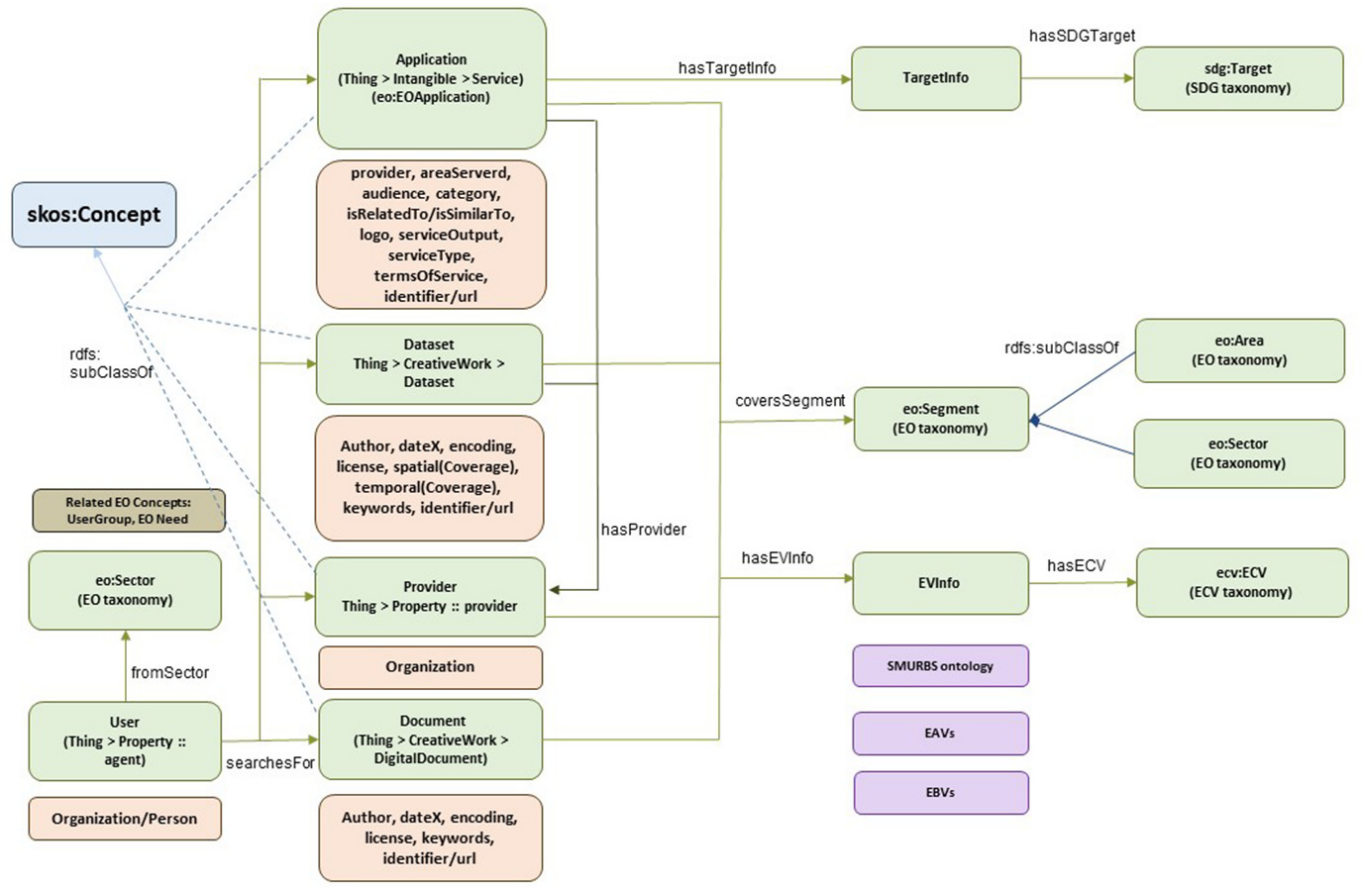
EIFF-O wrapper. This wrapper merges the EO ontology, the ECV ontology and the SDG ontology, also including concepts from schema.org in order to support JSON-LD data formats. In addition, through the concept of Essential Variables other ontologies can be linked (e.g., SMURBS, Essential Agriculture Variables and Essential Biodiversity Variables).

The EIFFEL Ontology supports the discovery of services and datasets from EO users. It consists of a few concepts that are linked with the ontologies described previously (EO, ECV, SDG) and is envisioned to be linked with other potential ontologies or concepts, considering the wide spectrum of EO data. An overview of the ontology is depicted in Figure 6, where the main concepts are related to the previous ontologies and are simultaneously associated with already existing concepts from *schema.org.*

A **user**, In the discovery phase, the user searches for four main items: (i) services or applications, (ii) datasets, (iii) providers, and (iv) documents. From schema.org’s available vocabularies, we can either use the *Organization* or *Person* concepts here to describe the *User.*

This user falls under a specific sector, a concept derived from the EO ontology/taxonomy, which defines 26 different sectors. Related to this concept, to further profile the user, one might add **user groups** and **EO needs** to the EO ontology.

One of the most interesting items to search for is the **applications** represents the highest level of usability beyond simple data. Such applications can be easily associated with the *Service* concept from schema.org as well as the *Application* concept from the EO taxonomy via an *rdfs:subClassOf* property. It can also be linked to the (input/output) datasets and the corresponding provider. Furthermore, the *Application* class is characterized by

- The **TargetInfo** class acts as a wrapper for encapsulating different targets or applications. A direct link is already specified with the SDG ontology by establishing a relationship with the *sdg:Target* concept.
- An **eo:Segment** class, which also acts as a wrapper to support the association with the EO ontology and includes two different perspectives (provider and market view), with **eo:Area** and **eo:Sector**, respectively.
- An **EVInfo** class is another wrapper that supports different Essential Variables. By default, the link with the Essential Climate Variables is provided via the **ecv:ECV** concept. Additionally, various links have been identified for inclusion in future work:
  - The **SMURBS ontology** also includes the ECV concept and extends it to *Essential Urban Variables* for smart city applications.
  - The **Essential Agriculture Variables** (EAVs) are specific variables that may be of interest for applications related to agriculture.
  - The Essential Biodiversity Variables (EBV) are another domain of significant research in the EO world.

**Datasets** are probably the most searched items, as the amount of available data is huge in the EO world, whereas applications tend to be local and not massively published. The dataset concept is already in *schema.org* but should probably be extended to cover specific EO aspects.

**Providers** are another way to search for data as they provide datasets, applications, or both. Currently, this class seems to map fairly well with the *Organization* concept in *schema.org* and supports the linkage between EO segments and EV information. Last but not least, **documents** are another category of data beyond datasets that can provide additional information about datasets and applications. The *Document* class is derived from the schema.org *DigitalDocument* concept, as some of their fields are useful.

## 4. EIFF-O supporting tools

Ontologies are essentially released as text-based OWL files (e.g., Turtle format), but the EIFFEL project goes a step beyond and provides a set of related software components called the EIFF-O module, which allows fast integration with other modular architectures using semantics from the EO domain.

**Figure 7.**
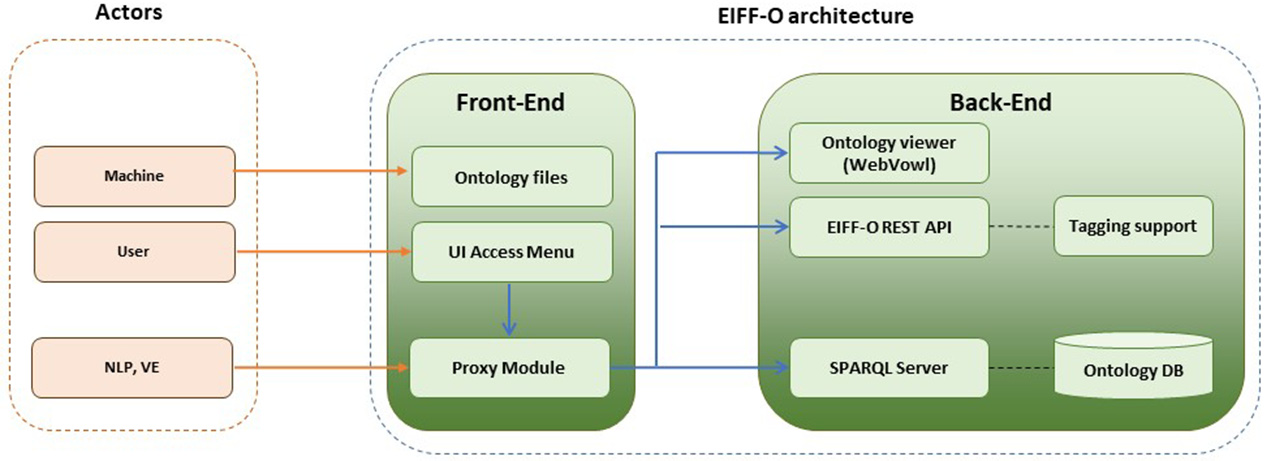
EIFF-O module (building blocks). The EIFF-O module consists of a series of tools that facilitate the understanding and usage of the EIFF-O ontology. There is a front-end to access and view ontology files, documentation and code, but also a backend part providing Webvowl support, a REST API as well as a SPARQL endpoint.

The EIFF-O module is composed of two main blocks: a front end and a back end, as shown in Figure 7. The front-end interacts with different external actors: (i) machines or software agents directly retrieving the different ontology files, (ii) users employing a graphical user interface, and (iii) other modules; for the EIFFEL project, they are the NLP cognitive search engine as well the Visualization Engine.

The back-end is responsible for providing services related to the supported ontologies:

•   The **ontology viewer** allows visual exploration of the different entities of the ontology as well as their properties and related instances. It is a web-based implementation of the Visual Notation for OWL ontologies (VOWL).

•   The **SPARQL endpoint** allows SPARQL queries for four different ontologies (EIFFO, EO, ECV, and SDG), which were previously uploaded to the SPARQL server. SPARQL is typically used in machines and software agents.

•   The **REST API** implements basic and specific functionalities to support other modules (for the EIFFEL project NLP and VE). First, it supports common requests to the ontology without the need to use SPARQL to provide a full JavaScript Object Notation (JSON) interface (e.g., obtain the list of SDG goals or targets as a JSON file). Second, it provides tagging support for different entities, mainly for datasets, but also for services and providers.

The architecture of the EIFF-O module is highly modular and is distributed in the form of Docker containers that allow fast deployment. The available GitHub repository includes all files with the code as well as a docker-composed file to use the related Docker containers.

## 5. EIFF-O usage in CC applications

The tagging of datasets with concepts from EIFFO is expected to bridge the gap between bare datasets and the development of applications for decision support systems. The ontology was evaluated in five pilots for the CC domain within the H2020 EIFFEL project: (i) Water and Land use management, (ii) Sustainable Agriculture, (iii) Transport Management, (iv) Sustainable Urban Development, and (v) Disaster Resilience.

Initial feedback was requested from the pilots to establish links with the developed ontology, as listed in Table 1.

**Table 1.** Summary table to collect semantic requirements per pilot. The table breaks down into several fields the way pilots can be described to provide later a set of relevant concepts, mainly related to Essential Climate Variables, that can later build a vocabulary and/or be incorporated as additional semantic metadata.

#	Title	Item	Information provided by pilot
**1**	General	Aim/scope	what is/are the current challenge(s) and what is the pilot doing to solve/analyze it/them?
		Link to CC	which aspects/areas of CC are being considered?
		Impact	what are the expected benefits?
**2**	Context	ECV _x_	**Scope:** *consumer/producer/both* **Relevance**: *critical/highly important/important/not important/mostly irrelevant* **Autonomy**: standalone/linked with other ECV **related vocabulary**: which related concepts/keywords do you associate with this ECVs?
**3**	GEOSS Data	Input _x_	**Name** **metadata** (link) **interface** (GEOSS portal/Copernicus/WEkEO/…) **discovery** (API) **relevance** *critical/highly important/important/not important/mostly irrelevant* **ECV** (relationship with ECVx, if any), **Other** * (any other aspect that you might consider relevant)*
		Output _x_	<name, metadata, interface, discovery, relevance, ECV, other>
**4**	Other Data	Input _x_	<name, metadata, interface, discovery, relevance, ECV, other>
		Output _x_	<name, metadata, interface, discovery, relevance, ECV, other>
**5**	Stakeholder	STH _x_	**Interaction Level (IL)**: active/passive user **Active Interaction Profile (AIP)**: (only for active users) Sample requests, outputs expected (with recommendations for linking with related data)

The feedback related to ECVs and GEOSS data was especially relevant, as they will serve as a basis for a comparison with the GEOSS portal. Involved stakeholders were also relevant in the framework of the project, as they sought the creation of Communities of Practice (CoPs) to enrich the developed CC applications within the pilots.

For the five pilots, different input variables (datasets) were identified and mapped to the ECVs and/or related terminology. An example is provided in the third pilot study.

- Input datasets in green are more relevant (they will be tagged with concepts from the ECV ontology), and pilots work with the dataset related to that ECV. At the end of each dataset, a mark was included (GCOS, ECV Inventory, GEOSS, Copernicus, Other) depending on whether pilots used the GCOS ECV website (e.g., for aerosols), ECV inventory, GEOSS portal, related Copernicus portals (including CAMS and DIAS), or other portals (local open/private portals) to discover the dataset.
- The input datasets in orange refer to those that were less relevant (they will probably not be tagged with concepts from the ontology) or pilots were not able to retrieve data to be used.

From Table 2, it is possible to tag the CC application either at *ECVs* or *ECVProducts level*, as they are both concepts in EIFF-O. Most users are likely to tag at the ECV level. However, when dealing with semantics, one may want to be as accurate as possible, so let us take the narrower concept of *ECVProduct*. Note that all products are instantiated as part of the released ontology, thus forming a knowledge base. For example, one may query the REST API to retrieve all products, one of which is the NO2 tropospheric column, as shown in the following JSON example.

**Table 2.** ECVs in EIFFEL and relation with Pilot 3. In order to assign a relevant concept from the ECV ontology, typically an Essential Climate Variable, a pilot is decomposed by inputs and outputs. From the inputs and outputs that relate to climate issues, they are listed in this table to check for potential mapping with one (or more) ECV concept.

**Transport Management**	**Inputs** **Atmosphere ➔ Surface ➔ Pressure (related product: pressure)** **Atmosphere ➔ Surface ➔Temperature (related product: Temperature)** **Atmospheric ➔ Surface ➔ Water vapour (related products: Total column water vapour, Tropospheric profiles of water vapour)** **[Source: CAMS and sentinel 5P]** **Atmosphere ➔ Surface ➔ Wind speed and direction (related product: Surface Wind Speed and Direction).** **[Source: CAMS and sentinel 5P]** **Atmosphere ➔ Atmospheric Composition ➔ Aerosols (related product: Aerosol-layer height)** **[Source: CAMS and sentinel 5P. PM2.5 is considered important, provided by CAMS. Sentinel 5P provides Aerosol Index]** **Atmosphere ➔ Atmospheric Composition ➔ Carbon dioxide, methane and other greenhouse gases (related product: Tropospheric CO2 column, Tropospheric CO2)** **[Source: CAMS and sentinel 5P]** **Atmosphere ➔ Atmospheric Composition ➔ Precursors for Aerosols and Ozone (related products: NO2 tropospheric column, SO2, HCHO tropospheric columns)** **[Source: CAMS and Sentinel 5P]** **Atmosphere ➔ Atmospheric Composition ➔ Ozone (related products: Total column ozone, Tropospheric Ozone)** **[Source: CAMS and sentinel 5P]** **Ocean ➔ Physical ➔ Ocean surface heat flux (related product: radiative heat flux)** **Ocean ➔ Physical ➔ Sea surface temperature (related product: Sea Surface Temperature)** **Ocean ➔ Physical ➔ Sea state (related product: wave height)** **Ocean ➔ Physical ➔ Sea level (related products: Global Mean Sea Level, Regional Mean Sea Level)** **Ocean ➔ Biogeochemical ➔ Ocean colour (related products: Water leaving radiance, Chlorophyll-a concentration)** **Ocean ➔ Biogeochemical ➔ Nitrous oxide (related product: interior ocean N20)** **(Expected) Outputs** **Atmosphere ➔ Atmospheric Composition ➔ Precursors for Aerosols and Ozone** **(Predicted parameters via ML: surface NO2, SO2)** **Atmosphere ➔ Atmospheric Composition ➔ Ozone** **(Predicted parameters via ML: surface Ozone)**


{
"oClass": "http:// purl.org/eiffo/ecv#ECVProduct" ,
"oInstance": "http://purl.org/eiffo/ ecv#ECVProduct_041",
"productDefinition": "Molecules of NO2 in the atmosphere from surface to tropopause (Molecules /cm2)",
"productName": "NO2 tropospheric column,”
"productRequirement": [
         {
         "frequency”: "4 hr ",
         "oClass": "http://purl.org/eiffo/ecv#ECVProductRequirement",
         "oInstance": "http://purl.org/eiffo/ecv#Requirement_041",
         "requiredMeasurementUncertainty" : "Max(20%,0.03DU) " ,
         " resolution": "5−10 km/NA" ,
         " stability ": "2%"
         }
  ]
}



The (REST) response contains all references to classes and instances within the knowledge base so that a further SPARQL query, if needed, is possible. The response also contains the associated ECVProductRequirement element; in this particular case, only one.

In this scenario, EIFF-O recommends tagging the CC application via a JSON-LD format:


{
"@context": " http://purl.org/eiffo/ecv",
"@type": "ECVProduct" ,
"productName": "NO2 tropospheric column"
}



Note that a permanent URL is used in the ECV ontology. In fact, the four different EIFF-O ontologies can be labeled or referenced independently, given by their prefixes in the implementation (the SDG ontology prefix has been retained):

**Table:** 

PREFIX ecv: < http://purl.org/eiffo/ecv#> PREFIX sdgo: < http://metadata.un.org/sdg/ontology#> PREFIX eo: < http://purl.org/eiffo/eotaxonomy#> PREFIX eiffo: < http://purl.org/eiffo#>

Clearly, further tagging is possible in the previous JSON-LD format with any other concept available in EIFF-O (e.g., SDGs). Although this is the recommended tagging format, other more simplistic approaches are possible if there is already another metadata format specification that does not support JSONLD. For example, in the previous example, by using a key-value approach, the following can be used:


{
" eiffo": "ecv ;ECVProduct ;NO2 tropospheric column".
} 



**Figure 8.**
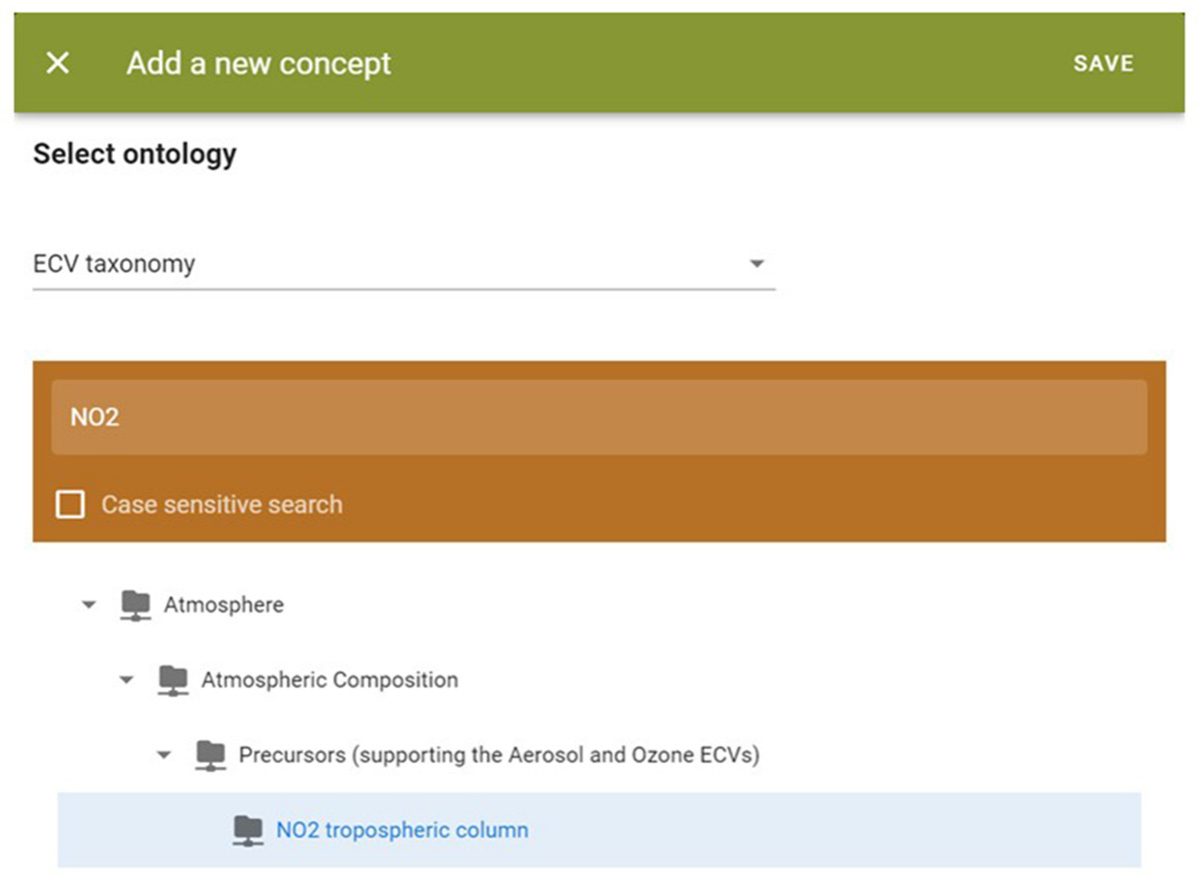
EIFF-O module (search for concept). During the tagging of datasets, and considering the ontologies used derived from taxonomies, users can easily find and select through tree-based structures the related concept to be associated to a specific dataset.

The EIFF-O module includes a demo tool to tag datasets with concepts from three ontologies (SDG, EO, and ECV). As the number of concepts is relatively high, the user first selects the taxonomy to be used and then the final concept; even in the second step, it is possible to search (only by name). Figure 8 shows an example of the ECVProduct ‘NO2 tropospheric column.’

**Figure 9.**
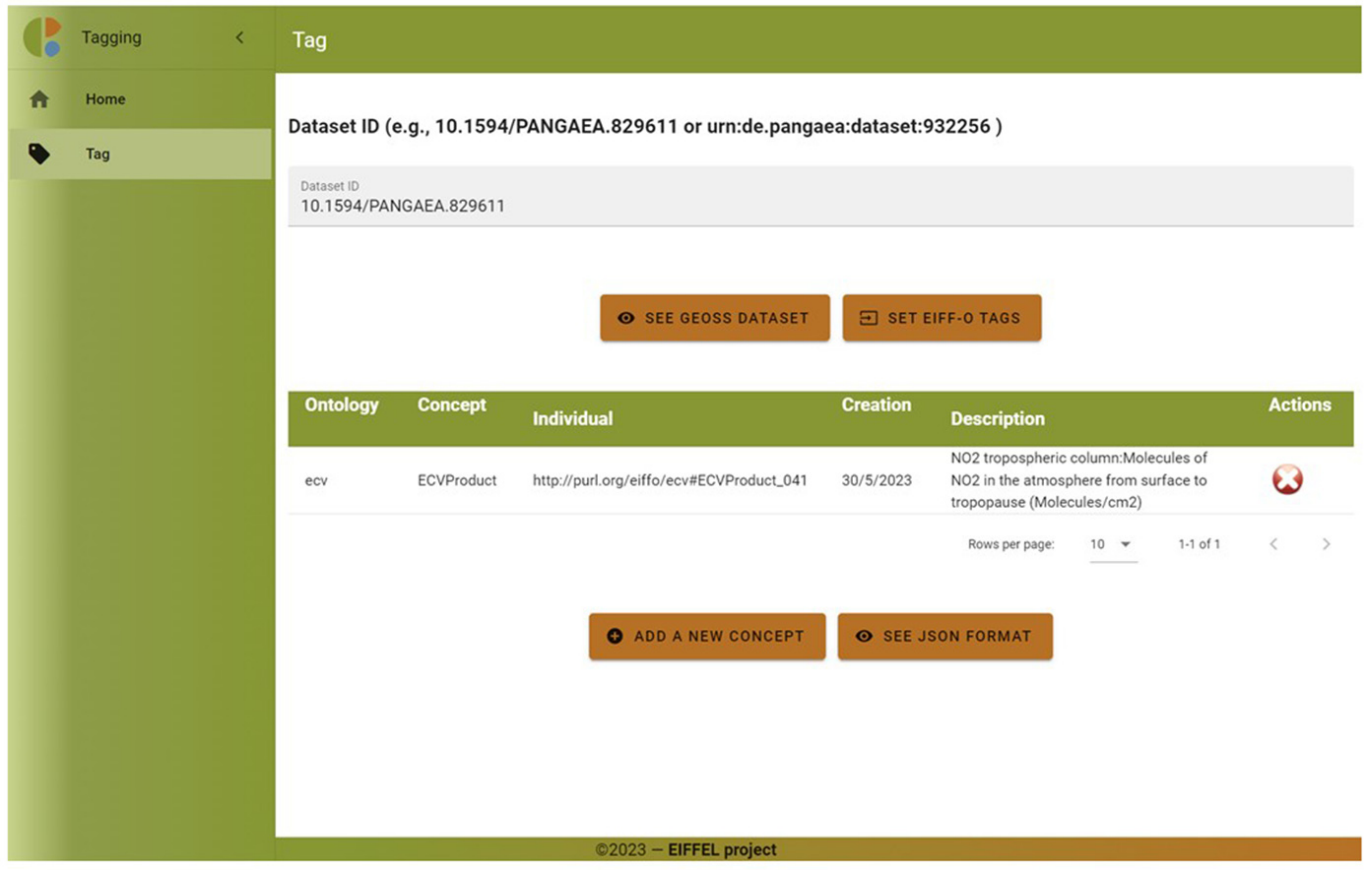
EIFF-O module (tagging visual table). The tagging tool allows the user to easily list all semantic concepts from EIFF-O associated to a certain dataset. They are listed in a table describing the related concept and some actions to be performed (add concept, delete concept).

After clicking on the ‘Save’ button, the concept is added to a visual table (see Figure 9). The user can add additional concepts either from this taxonomy (ECV) or other taxonomies (EO, SDG) if applicable. Additionally, the user has the option to view the data from the table in JSON format.

## 6. Conclusions

This paper presented an ontology (and knowledge base) in a modular manner composed of four different ones: the ontology is enriched with a set of supporting tools, as well as a clear online documentation portal (readthedocs ^20^) and open source repositories (GitHub ^16^, DockerHub ^21^). An online demo ^22^ and a YouTube tutorial ^23^ are also available.

EO taxonomy, only specified by EARSC, has been implemented and provides a general perspective. The current specification studied the user and provider perspectives and established various classifications. This paper provides a valid implementation of this specification and also allows updating of the ontology as new categorizations appear in the future, as well as enhancing or further refining any of the currently implemented sectors and/or areas.

The ECV taxonomy, only specified by the GCOS, has also been implemented and provides a specific perspective. In fact, information is only provided on a website, and some formalism is missing. The taxonomy includes a two-level hierarchy (atmosphere, land, and ocean with their own subcategories), as well as product-related concepts. During the writing of this paper GCOS updated the taxonomy with minor changes at ‘ECVProductRequirement’ level, ECVs and categories (domains) remain. The authors of this paper strongly believe that enriching the contact and support information related to ECV stewards would probably accelerate the use of available ECV datasets. Moreover, the ECV data sources specified by the GCOS are a part of the ECV knowledge base. This allows automatic tagging of the GEOSS datasets by mapping the associated metadata.

The UN’s SDG ontology (and knowledge base) have been integrated as part of the EIFF-O, providing a goal-oriented perspective. It embeds SDG ontology, as its concepts are essential for categorizing or classifying current and future applications, both from research and political perspectives. There is ongoing research using EO data and models to support SDGs ^24^, which represents another future research line to track.

Some vocabularies from Schema.org have been included to publish EO datasets and applications in this format and promote the usage of the semantic web, which will allow semantic search engines to harvest data directly from the provider’s website without the need for any intermediate broker. In this regard, the GEOSS Data Access Broker is only temporarily necessary to support this transition. Finally, examples of integration with applications and tools are provided. EIFF-O supported the CC applications developed in the EIFFEL project, as well as the NLP engine and the visualization engine, to enrich the user experience, which was also an additional work, but outside the scope of this paper.

## Data Availability

Zenodo: Report on the EIFFEL Ontology ,( https://doi.org/10.5281/zenodo.7852156 ^25^. This project contains the following underlying data: An extended report for the EIFFEL ontology is available in Zenodo because of a task within the EIFFEL project (public deliverable).

## Software availability

Source data encompassing the different ontologies is available:

- GitHub: https://github.com/benmomo/eiffel-ontology. The repository includes information about usage, documentation, and licenses.

Besides the ontology, the EIFF-O tools can be deployed through:

- A docker-compose file available in GitHub https://github.com/benmomo/eiffel-ontology ^16^ ,which makes use of
- Docker images available in DockerHub https://hub.docker.com/u/benmomo ^21^ .

Online documentation is available via ReadtheDocs https://eiffel-ontology-doc.readthedocs.io/en/latest/ ^20^

If you would like to skip some required configuration for the docker-compose, there is also

- Online demo https://eiffo.satrdlab.upv.es/ ^22^, where users can visualize the ontology, launch the tagging tool, etc.
- An online YouTube tutorial https://youtu.be/4maOkmRMnNE?si=i5wzJZAwOqz2GK5p ^23^

### Extended data

- The datasets used for tagging have been mainly extracted from the GEOSS DAB available in https://www.geodab.net/ ^26^
