# Supplementary material for: Enriching Earth observation datasets through semantics for climate change applications: The EIFFEL ontology
Source: Open Res Eur. 2024 Oct 18;4:133. Originally published 2024 Jul 2. [Version 2] doi: 10.12688/openreseurope.17992.2 (PMC11555329; doi:10.12688/openreseurope.17992.2)
Supplement: Peer review history

# Reviewer report: Reviewer response for version 2

**DOI:** 10.21956/openreseurope.20265.r46316

**Published:** 2024-12-23

**Authors:** Despina-Athanasia Pantazi

**Affiliations:** 1 National and Kapodistrian University of Athens, Athens, Greece

In this paper, the authors introduced the EIFFEL ontology (EIFF-O), which incorporates the Earth Observation (EO taxonomy), the Essential Climate Variables (ECVs) ontology, the Sustainable Development Goals (SDGs) ontology, and the EIFF-O wrapper. Their goal is to facilitate better integration of EO datasets for the data providers and the users. Moreover, they enriched EIFF-O with a set of supporting tools to assist its utilization, and they promoted the usage of the semantic web by including some vocabularies from Schema.org to publish EO data. They evaluated the ontology in five pilots for the climate change domain. 

 Overall, this is a well-written paper which tackles the challenge of discovering EO datasets due to inconsistent metadata availability. The figures, the online documentation and the demo are quite helpful for better understanding the ontologies, and the usage of the Schema.org will enhance the discovery of the EO datasets through the major search engines. 

 There are some minor mistakes and comments for the authors to consider in order to improve their paper:

1. The authors included one dataset concerning the “Atmospheric NO2 Levels”, but there is only one concept tagged. It would be very useful to have one more detailed specific dataset annotated with the proposed ontology, that includes the relevant tags for its available properties as well. Even though the classes and the properties of each ontology are defined in the paper, it would be very helpful to describe the available metadata of a specific dataset and map it to the appropriate components of the presented ontology as well. The example can include the appropriate Schema.org annotations as well.
2. In various sections of the paper, the authors provide a number of entities (e.g., page 7: “31 different areas”, page 8: “80 services in different areas”, page 9: “31 ECV steps involved”, etc). It would be useful to provide some examples of these entities for the reader to better understand each group of entities and some references to the full lists.
3. Minor errors: page 10: “A user, In the discovery”, “... is the applications represents…”
4. The references for the external vocabularies UNBIS and Eurovoc are missing.

Is the study design appropriate and does the work have academic merit?

Yes

Is the work clearly and accurately presented and does it cite the current literature?

Yes

If applicable, is the statistical analysis and its interpretation appropriate?

Not applicable

Are all the source data underlying the results available to ensure full reproducibility?

No source data required

Are the conclusions drawn adequately supported by the results?

Yes

Are sufficient details of methods and analysis provided to allow replication by others?

Yes

Reviewer Expertise:

Semantic Web, Earth Observation, Natural Language Processing, Machine Learning

I confirm that I have read this submission and believe that I have an appropriate level of expertise to confirm that it is of an acceptable scientific standard.

# Reviewer report: Reviewer response for version 2

**DOI:** 10.21956/openreseurope.20265.r45285

**Published:** 2024-12-19

**Authors:** Antonis Troumpoukis

**Affiliations:** 1 National Centre for Scientific Research “Demokritos”, Agia Paraskevi, Greece

The authors have addressed my comments reasonably well. I think the paper should be accepted.

Is the study design appropriate and does the work have academic merit?

Yes

Is the work clearly and accurately presented and does it cite the current literature?

Yes

If applicable, is the statistical analysis and its interpretation appropriate?

Not applicable

Are all the source data underlying the results available to ensure full reproducibility?

No source data required

Are the conclusions drawn adequately supported by the results?

Partly

Are sufficient details of methods and analysis provided to allow replication by others?

Partly

Reviewer Expertise:

Knowledge Graphs, Semantic Web, Earth Observation, Geospatial data

I confirm that I have read this submission and believe that I have an appropriate level of expertise to confirm that it is of an acceptable scientific standard.

# Reviewer report: Reviewer response for version 2

**DOI:** 10.21956/openreseurope.20265.r46318

**Published:** 2024-12-19

**Authors:** Raul Palma

**Affiliations:** 1 Poznan Supercomputing and Networking Center, Poznan, Poland

The paper presents the EIFFEL ontology, comprised of four individual ontologies, and a set of supporting tools to use/visualise the ontologies. After the introduction, the paper introduces the methodology followed to construct the ontologies, and then describes the ontologies and supporting tools. The paper is generally easy to read and provides links to access the generated resources (ontologies, tools). 

 There are, however, several issues. 

 * The paper probably misses various existing works that are relevant to the presented work. For example, there are various EO portals/catalogues that support/use formal classifications (taxonomies) in their metadata, e.g., [1], [2] . Also, regarding ECV there are ongoing efforts (e.g., [3], [4])

 * The paper claims that  EIFF-O supported the CC applications developed in the EIFFEL project, as well as the NLP engine and the visualisation engine to enrich the user experience. This claim cannot verified at all. Paper says that this was an additional work, but outside the scope of this paper. So, either provide reference to where this is described and available or remove this claim. 

 Regarding ontologies:

 It is mentioned that EIFFO ontology plays a role of wrapper that brings together the three sub-ontologies (EO, ECV, and SDG). However, the ontologies are not really properly connected: 

 * EIFFO ontology does not import the 3 sub-ontologies to put all of them together. As it is now, EIFFO ontology does not have much knowledge about the terms specified in EO ontology or ECV ontology or SDG ontology. For example, EIFFO reuses some concepts from EO but does not import those concepts definitions to be exploited in the target application, e.g., both Sector and Segment are imported but their relationship (Sector subclass of Segment) is not. 

 Even if the target applications import individually all the ontologies, there are various conflicts/inconsistencies, e.g., :

 * the ECV class is defined in EO ontology (http://purl.org/eiffo/eotaxonomy#ECV) and EIFFO ontology (imported from ECV ontology) (http://purl.org/eiffo/ecv#ECV), differently although apparently they refer to the same concept. 

 * Domain class is defined both in in EO ontology (http://purl.org/eiffo/eotaxonomy#Domain ) and ECV ontology ontology ( http://purl.org/eiffo/ecv#Domain), differently although apparently they refer to the same concept. 

 * fromSector object project is defined both in EO ontology (http://purl.org/eiffo/eotaxonomy#fromSector) and EIFFO ontology ( http://purl.org/eiffo/ecv#fromSector), and differently

 * hasDomain object property is defined both in ECV ontology ( http://purl.org/eiffo/ecv#hasDomain) and EO ontology (http://purl.org/eiffo/eotaxonomy#hasDomain), differently

 * domainName data type property is defined both in EO and ECV ontologies

 Other issues in ontologies:

 * In ECV ontology, you have some typos that created multiple properties,, i.e., hasECVProducRequirement and hasECVProductRequirement, scientificAreaName and scientifiAreaName. 

 * In EO ontology, you have typo in SKOS namespace definition: @prefix skos: , thus skos:altLabel becomes http://www.w3.org/2004/02/skos/corealtLabel, skos:broader is defined as object property and  http://www.w3.org/2004/02/skos/core#broader as annotation property….

 * Note also that SDG ontology (which you only reuse) has various issues, e.g., change definition of imported terms, e.g., using the chain property axiom on schema:keywords (which is defined as an rdf:Property) you are actually creating two properties, one datatype property and one object property. It will be usable but probably is not what you wanted? Also according to OWL punning, OWL 2 [5] DL still imposes certain restrictions: it requires that a name can only be used for one kind of property, so this would become an OWL 2 full ontology (see also [6]). But most importantly this is not how schema:keywords is defined. Similarly dcterms:subject (also an rdf:Property) is imported but redefined with domain/ranges. If there is a need to define additional constrains, sub-properties may be defined.

 Additional comments about modelling choices

 * EIFFO defines another class Application as subclass of EOApplication in EO ontology. Application class is defined as subclass of schema:Service, skos:Concept and EOApplication; but EOApplication is already subclass of schema:Service and skos:Concept (knowledge it can get if it imports EO ontology).  EIFFO associates Application class with various properties, but is there any particular reason not to extend directly EOApplication? 

 * It seems there is a predefined set of data sources types, why don’t you create a code list, which would allow you to describe and refer to these values (individuals), and then associate the datasource with this code list via a property similar to dstype, 

 * The way requirements are modelled in ECV ontology is worth reconsider. Currently, all products individuals have only one associated requirement individual, which is defined with the real requirements as simple strings, e.g., for frequency, requiredMeasurementUncertainty, resolution, etc. Perhaps, may be useful to define separated the individual requirements, thus defining them once and reused by multiple products. This could have multiple benefits including avoiding typos inconsistencies, e.g., defining :resolution "Site" or :resolution “sites”. 

 * You mention OWL 2 EL is the most suitable choice for EIFFEL ontology, because it is designed for ontologies that emphasise hierarchical classification and require efficient reasoning over large datasets. Indeed such profile has typical use cases with large classifications like SNOMED-CT with thousands of concepts and/or large number of properties, which does not seem the case in EIFFEL. In any case, is not clear what kind of modelling choices you made to adhere to OWL 2 EL? In fact, there seems to be some violations of the OWL 2 EL profile specification (you can check using e.g., [7])

 Regarding EIFFO usage

 * Authors mention that in the example presented below table 3, EIFF-O recommends tagging the CC application via some JSON-LD. This is not clear, where is this recommendation provided? Which CC application? 

 * Moreover, the JSON-LD extract provided is not valid. The "@context": " http://purl.org/eiffo/ecv" is incorrect, i.e., it’s not pointing to any JSON-LD context, which maps terms to IRIs (from the ontology) (see [8]). Consider using a validator for your JSON-LD documents/payloads (e.g., [9])

 * Additionally, it’s not clear why the demo annotation tool is not producing JSON-LD as authors claim to promote. Also, the JSON generated is only providing a set of JSON nodes with some undefined attributes and there is no link with the dataset id provided…. 

 —

 Other minor issues:

 * Consider improving ontology diagrams. Please consider creating more standard/consistent diagrams for ontologies (e.g., [10] or in UML like fashion [11])

 ** Paper mentions that blue arrows represent ‘rdfs:subclassOf’, which denotes the relationship with SKOS, a common data model for building taxonomies. However, subclassOf is not only denoting relationship with SKOS

 ** Paper says that brown boxes (which actually seem orange) denote data properties for the nearby class (green boxes). 

 Brown boxes seem to show name of individuals in EO ontology figure. In ECV ontology in one case represent possible values of one data property and in another case the data properties associated to the class. In SDG ontology it represents also values of an (object) property. In EIFFO ontology it’s really not clear at all what is inside the brow boxes (what is dateX, areaServed? etc.) 

 ** Paper says pink boxes (which actually seem Violet) denote a potential relationship with other subontologies through a nearby class (green box).. What kind of relationship?

 ** What about blue boxes?

 * The EIFF-O module separation of front-end and back-end is not very intuitive, i.e., how an ontology visualisation interface is a back-end component? 

 * The front-end components are not described, e.g., there is no information what is the proxy module

 * You have good documentation of the work, but it would be useful to have a more technical documentation of the ontologies themselves, e.g., [12] or [13] (which also includes VOWL)

 * Why table 3 is not providing the datasets using the metadata proposed in table 2 (i.e.. name, metadata, interface, discovery, relevance, ECV, other> ? In table 3, it is mentioned that at the end of each dataset, a mark was included depending on whether pilots used specific source. In the example it seems you only include this information for datasets coming from CAMS and sentinel 5P. Can you provide other examples and/or statistics based on the source. Also in table 3 perhaps is not needed the first column if you just mention these datasets are from pilot transport management. 

 * The (REST) API cannot be used/tested because require authentication, and the demo SPARQL endpoint is not provided.

 * In the demo annotation tool, the button to see GEOOS dataset does not seem to work (at least for examples)

 * In introduction, in “semantic versus semantic interoperability”, do you mean syntactic vs semantic interoperability?

 *The EO taxonomy ontology namespace (http:/purl.org/eiffo/eotaxonomy ) is not resolving

 [1] https://fedeo.ceos.org/intro.html

 [2] https://fedeo.ceos.org/features.html#id9

 [3] https://www.rd-alliance.org/groups/interoperable-descriptions-observable-property-terminology-wg-i-adopt-wg/wikis/?wiki=136935

 [4] https://defs-dev.opengis.net/vocprez-hosted/object?uri=https%3A//w3id.org/ad4gd/ev/ecv

 [[5] https://www.w3.org/TR/2012/REC-owl2-new-features-20121211/#F12:_Punning

 [6] https://stackoverflow.com/questions/49570256/what-are-the-consequences-of-declaring-a-property-subpropertyof-a-dublincore-pro

 [7] https://github.com/stain/profilechecker

 [8] https://www.w3.org/TR/json-ld/#the-context

 [9] https://json-ld.org/playground/

 [10] https://coe.qualiware.com/templates/ontologydiagram-owl/

 [11] https://kg.ontotext.com/metaphactory/resource/Help:VisualOntologyEditing#diagram-canvas, https://protegewiki.stanford.edu/wiki/OWL2UML

 [12] https://essepuntato.it/lode/

 [13] https://github.com/dgarijo/Widoco

Is the study design appropriate and does the work have academic merit?

Partly

Is the work clearly and accurately presented and does it cite the current literature?

Partly

If applicable, is the statistical analysis and its interpretation appropriate?

Not applicable

Are all the source data underlying the results available to ensure full reproducibility?

Yes

Are the conclusions drawn adequately supported by the results?

Partly

Are sufficient details of methods and analysis provided to allow replication by others?

Yes

Reviewer Expertise:

ontology engineering, semantic technologies, linked data

I confirm that I have read this submission and believe that I have an appropriate level of expertise to state that I do not consider it to be of an acceptable scientific standard, for reasons outlined above.

# Reviewer report: Reviewer response for version 2

**DOI:** 10.21956/openreseurope.20265.r45286

**Published:** 2024-11-11

**Authors:** Victoria Lush

**Affiliations:** 1 Aston University, Birmingham, England, UK

I would like to thank the authors for detailed review responses. I have no further issues with the article.

Is the study design appropriate and does the work have academic merit?

Yes

Is the work clearly and accurately presented and does it cite the current literature?

Yes

If applicable, is the statistical analysis and its interpretation appropriate?

Not applicable

Are all the source data underlying the results available to ensure full reproducibility?

No source data required

Are the conclusions drawn adequately supported by the results?

Yes

Are sufficient details of methods and analysis provided to allow replication by others?

Yes

Reviewer Expertise:

GIS, Earth Observations, data analytics, conceptual semantic interoperability.

I confirm that I have read this submission and believe that I have an appropriate level of expertise to confirm that it is of an acceptable scientific standard.

# Reviewer report: Reviewer response for version 1

**DOI:** 10.21956/openreseurope.19446.r43918

**Published:** 2024-10-17

**Authors:** Antonis Troumpoukis

**Affiliations:** 1 National Centre for Scientific Research “Demokritos”, Agia Paraskevi, Greece

This paper presents the EIFFEL ontology (EIFF-O), a comprehensive and modular ontology designed to enhance the organization, discoverability, and interoperability of Earth observation (EO) datasets. The ontology incorporates taxonomies for EO services, Essential Climate Variables (ECVs), and Sustainable Development Goals (SDGs), providing a framework for classifying and linking EO data. The authors also provide supporting tools and resources, including an ontology viewer, SPARQL endpoint, REST API, and online documentation, to facilitate the adoption and utilization of EIFF-O. The ontology's practical value is demonstrated through its evaluation in five climate change pilot pilots.

**Strengths:**

- Addresses a Critical Need: The paper effectively addresses the significant challenge of inconsistent metadata and poor organization in EO datasets, which hinders effective data discovery and use.
- Comprehensive Approach: The modular design of EIFF-O, incorporating EO, ECV, and SDG taxonomies, provides a holistic framework for classifying and linking EO data from various perspectives.
- Semantic Web Integration: The use of schema.org vocabularies and JSON-LD formats promotes semantic web integration, enhancing data discoverability and interoperability.
- Practical Tools and Resources: The supporting tools, APIs, and online resources make EIFF-O accessible and user-friendly for developers and researchers.
- Real-World Applicability: The evaluation of EIFF-O in climate change pilot projects demonstrates its practical potential in addressing real-world challenges.

**Minor Revisions/Reservations:**

- Non-aligned Methodology Steps: The "Methods" section mentions a seven-phase methodology but only mentions the first five and details the second to fourth. Including the remaining phases would provide a more complete picture.
- Need for a Concrete Example: While the paper explains the ontology's components well, it lacks a full concrete example demonstrating its application on a specific dataset. One (or more, with varying details) concrete example might show tagging it with ECV terms and relevant SDG targets, thus making the approach easier to grasp for readers.
- Potential for Redundancy: The integration of multiple taxonomies could lead to some overlap or redundancy in concepts, potentially causing confusion. For instance, the EO taxonomy's "Environment and Climate" market could have elements related to climate change that also appear in the ECV taxonomy. A paragraph addressing how these issues were or can be prevented or checked would strengthen the paper.
- Evaluation of Pilot Projects: Section 5 of the paper provides a hint that an evaluation or assessment of the ontology was performed, although it doesn't explicitly detail the process. More information on how the ontology was assessed would strengthen the claims.

**Overall Assessment:**

 This paper makes a valuable contribution to the field of EO data management by providing a well-structured and comprehensive ontology Addressing the minor revisions outlined above would further strengthen the paper and enhance its impact.

**Recommendation: **Approved with Reservations

Is the study design appropriate and does the work have academic merit?

Yes

Is the work clearly and accurately presented and does it cite the current literature?

Yes

If applicable, is the statistical analysis and its interpretation appropriate?

Not applicable

Are all the source data underlying the results available to ensure full reproducibility?

No source data required

Are the conclusions drawn adequately supported by the results?

Partly

Are sufficient details of methods and analysis provided to allow replication by others?

Partly

Reviewer Expertise:

Knowledge Graphs, Semantic Web, Earth Observation, Geospatial data

I confirm that I have read this submission and believe that I have an appropriate level of expertise to confirm that it is of an acceptable scientific standard, however I have significant reservations, as outlined above.

# Response

**Published:** 2024-10-29

**Authors:** Benjamin Molina

**Affiliations:** Communications, Universitat Politecnica de Valencia, Valencia, Valencian Community, Spain

We appreciate Reviewer 3’s thorough and insightful feedback on our manuscript. This third review arrived shortly after we had submitted an updated version of the manuscript, which included responses to comments from the first two reviewers. Authors are encouraged to refine their work based on at least two sets of reviewer insights before publication. Accordingly, we anticipate that part of Reviewer 3’s observations will already be addressed in this updated version. Nonetheless, we recognise that further clarification is warranted for some specific areas highlighted by Reviewer 3. The responses below aim to address these points, providing additional context and specific examples to enhance understanding of the manuscript’s scope, methodology, and practical applications. We appreciate the reviewer’s input in refining the manuscript and strengthening the presentation.

 Responses to the comments:

 1) *The first two reviewers already noticed this, and it has been updated in the new version of the paper. Now, there is one added short paragraph for each step (constraints and instances)*

   2) *The example we provide in the paper involves a dataset concerning “Atmospheric NO2 Levels.” Any dataset coming from GEOSS, including such information (or at least closely related to it), should be valid. Unfortunately, it is difficult to find such information in the GEOSS portal most of the time, and data scientists need to perform the tagging afterwards once the dataset has been found.* *For this paper’s dataset, we use ontology to assign relevant Essential Climate Variable (ECV) and Sustainable Development Goal (SDG) tags to illustrate how they can be contextualised within climate change applications.*

***Tagging with ECV Terms:***

*The dataset is tagged under the "NO2 tropospheric column" concept within the ECV taxonomy, specifically classified within the domain “Atmosphere > Atmospheric Composition.” This classification is shown in Figure 9 and enables users to locate datasets relevant to atmospheric NO2 concentration specifically, facilitating efficient discovery for users focused on climate change metrics.*

***Tagging with SDG Terms:***

*The process is analogous if the data scientist (typical user here) wants to tag the dataset with a concept from the SDG ontology. First, they select the SDG ontology, and then the goal can be selected (E.g., goal 13- climate action). There is no need to select a specific target within the goal; this is up to the user.  *  

 3) *This is a common situation when dealing with various ontologies, that some concepts might overlap, and one has to decide which one to take or even redefine the concept, establishing the links with the original concepts. However, this is a further step if EIFF-O gets widely accepted and evolves. EIFF-O proposes to start with these three taxonomies and identify which categories and concepts are more relevant based on usage.* *Currently, there is no overlap between the “Environmental and Climate” from the EO taxonomy and ECV’s taxonomy: the former does not include further concepts under this category, in fact, EARSC (owner of EO’s taxonomy) intended to extend it but didn’t, in its latest version.* *In general, there is no overlap in concepts so far with the three taxonomies *  

*4)* *The ontology is not directly assessed in the five pilots described in section 5 but rather in an indirect way. To bring more clarity to the context of the project:*

- *The five pilots had already identified their input datasets when EIFF-O was developed. At this phase, they could tag some of their datasets with concepts from EIFF-O, as described in the paper. This will increase the properties of those datasets and will potentially facilitate the discovery for future researchers*
- *EIFF-O was integrated as part of the EIFFEL horizontal tools, which include a semantic/cognitive discovery tool and a visualisation engine. This was left outside the scope of this paper, as the associated deliverables were not open (publicly available)*
- *The cognitive search engine (* *https://github.com/LIBRA-AI-Tech/cognitive-search-engine* *) extends GEOSS broker data with additional properties, one of them EIFF-O tags, as described in the paper. The visualisation engine allows (through the cognitive search API) the search and filtering of EIFF-O datasets by EIFF-O tags. *
- *Users from the five pilots tested the cognitive search engine and compared it with the GEOSS Portal, giving feedback on it in a subjective way. It was difficult to achieve objective metrics, as it would require pre-existing tagging of EIFF-O concepts in advance.*

# Reviewer report: Reviewer response for version 1

**DOI:** 10.21956/openreseurope.19446.r43925

**Published:** 2024-10-04

**Authors:** Victoria Lush

**Affiliations:** 1 Aston University, Birmingham, England, UK

This paper presents the EIFFEL ontology (EIFF-O) and the supporting tools developed by the authors. The authors’ motivation for introducing EIFF-O is that, despite the recognised need for semantic search and discovery of Earth Observation data, applications, and services, to date, no major aggregation portals offer semantic search functionality.

 EIFF-O incorporates: the Earth Observation (EO) taxonomy, the Essential Climate Variables (ECVs) ontology, the Sustainable Development Goals (SDGs) ontology, and the EIFF-O wrapper. The ontology is designed in a modular way to support further development and extensions. The authors additionally evaluate the developed ontology in five pilots for the climate change domain.

 Overall, the paper is well-written and presents a valuable contribution to the Earth Observation domain.

 There is a small number of concerns that the authors might want to consider addressing - 

 The authors use a 7-step methodology to design the EIFFEL ontology, however only 5 steps are described in detail in the manuscript. Constraints and Instances stages are not described – were these not applied when designing EIFF-O?

 On page 11, “ *Ontologies are essentially released as text-based OWL files (e.g., Turtle format)*” – it would be beneficial to briefly introduce OWL or offer a citation link.

 On page 11, “ *The EIFF-O module is composed of two main blocks…*” – perhaps provide a URL to the EIFF-O module demo here as well?

 On page 11, “ *The ontology viewer allows visual exploration of the different entities of the ontology*…” – when reading, I was wondering if there is a URL to the Viewer tool or is this tool available for deployment on GitHub? Only after getting to the end of the paper, I found all the links. It would be useful to provide the references where the tools are mentioned for the first time.

 Same as for the Viewer Tool, the SPARQL endpoint and the REST API could have a reference to the corresponding URLs.

 I found section “ *5. EIFF-O usage in CC applications*” somewhat confusing. I appreciate that it was kept brief, but is it challenging to understand how the ontology was actually evaluated in five pilots. Perhaps there is a supporting report that can be cited here for further reading?

 On page 12, “Input datasets in green…” and “The input datasets in orange…” – what figure do these refer to? I found this part confusing.

 On page 13, “ *The EIFF-O module includes a demo tool to tag datasets with concepts from three ontologies*…” – the authors offer screenshots of the tagging tool, but not of the other tools they developed. It would be beneficial to include a screenshot of at least the Viewer tool as well.

 On Page 16, “Online demo https://eiffo.satrdlab.upv.es/22, where users can visualize the ontology, launch the tagging tool, etc” – I tried this link using Firefox and only GitHub repository button worked. I was able to open other links in Chrome, but not Firefox. I wonder if other readers might encounter the same problem.

Is the study design appropriate and does the work have academic merit?

Yes

Is the work clearly and accurately presented and does it cite the current literature?

Yes

If applicable, is the statistical analysis and its interpretation appropriate?

Not applicable

Are all the source data underlying the results available to ensure full reproducibility?

No source data required

Are the conclusions drawn adequately supported by the results?

Yes

Are sufficient details of methods and analysis provided to allow replication by others?

Yes

Reviewer Expertise:

GIS, Earth Observations, data analytics, conceptual semantic interoperability.

I confirm that I have read this submission and believe that I have an appropriate level of expertise to confirm that it is of an acceptable scientific standard, however I have significant reservations, as outlined above.

# Response

**Published:** 2024-10-15

**Authors:** Benjamin Molina

**Affiliations:** Communications, Universitat Politecnica de Valencia, Valencia, Valencian Community, Spain

Dear reviewer, Many thanks for the review and comments. Below, you can find my response to the action items:  

1. (2-steps missing in the methodology) The first reviewer already noticed this, and it has been updated in the new version of the paper. Now, there is one added short paragraph for each step (constraints and instances)
2.  (OWL brief introduction) The document has been updated at the beginning of chapter 4, including a brief description and a link as a citation.
3. (Page 11 issues) I clarified at each point in the paper that all links are provided at the end, as this is the structure to follow in the published papers, according to the template.
4. (EIFF-O usage in CC applications) The ontology is part of a larger tool within the EIFFEL project, covering the visualisation and cognitive search engine. However, the last tools were considered confidential within the project; thus, the associated deliverables are not public. The cognitive search engine allows better mapping of the datasets available under GEOSS with the input text while searching. Adding additional metadata from EIFF-O also facilitates the future search. Thus, pilots (and any other researcher) can tag GEOSS datasets with EIFF-O concepts. The cognitive search engine was shown to relevant GEO representatives and was well accepted.
5. (Page 12 issue) Green and orange datasets refer to Table 2, not to any figure. Added in the text to not confuse anyone
6. (Page 13 issue) This was also noticed by the first reviewer. A screenshot of the Webvowl tool is included in the paper in section 4, when the ontology viewer appears for the first time. By the way, the online documentation in readthedocs includes screenshots of the tool
7. (Page 16 issue) The online demo is at https://eiffo.satrdlab.upv.es/, and the front-end HTML page was developed for Google Chrome; you are right. Some links might not work in other browsers. This notice is added in the paper, as well as the link to the services, so that they are reachable through other browsers:

# Reviewer report: Reviewer response for version 1

**DOI:** 10.21956/openreseurope.19446.r41946

**Published:** 2024-08-26

**Authors:** Guohui Xiao

**Affiliations:** 1 University of Bergen, Bergen, Norway

The paper presented the EIFFEL ontology (EIFF-O) for Earth Observation (EO) datasets. The creation of EIFF-O follows a methodology of 7 steps (from scope to instances).The resulted ontology consists of four components: the EO taxonomy, the ECV taxonomy, the DSG taxonomy, and the EIFF-O wrapper. There are also supporting tools developed by the EIFFEL project. The usage of EIFF-O is demonstrated in a climate change case. The paper is mostly well-written, and easy to follow. It is a nice contribution of the area of earth observation area.

 There are some minor recommendations for improving the papers:

 1. In Section 2, the methodology consists of seven steps, but the only the first five are discussed in the text.

 2. The statistics of the ontology (e.g., numbers of classes and properties) of each module should be provided.

 3. The expressiveness/profile of the ontology (ie. OWL 2 QL, EL, RL, DL) should be discussed

 4. The ontologies are available in the Github repository. However, the URIs of the ontology are not working .There is one single permanent URI of the ontology: https://purl.org/eiffo, which is not working. Please fix it. It would be also useful to provide permanent URIs for different modules. This is particularly relevant because two separate license (Apache 2 and CC BY 4.0) are used for different modules.

 5. In Section 4, it is useful to provide screenshot of the ontology viewer. What is the technology behind the technology? Moreover, is the SPARQL endpoint used only with the ontology, or there are also instance data to play with?

Is the study design appropriate and does the work have academic merit?

Yes

Is the work clearly and accurately presented and does it cite the current literature?

Yes

If applicable, is the statistical analysis and its interpretation appropriate?

Not applicable

Are all the source data underlying the results available to ensure full reproducibility?

Partly

Are the conclusions drawn adequately supported by the results?

Yes

Are sufficient details of methods and analysis provided to allow replication by others?

Partly

Reviewer Expertise:

Ontology, Semantic Web, Knowledge Graphs

I confirm that I have read this submission and believe that I have an appropriate level of expertise to confirm that it is of an acceptable scientific standard, however I have significant reservations, as outlined above.

# Response

**Published:** 2024-10-15

**Authors:** Benjamin Molina

**Affiliations:** Communications, Universitat Politecnica de Valencia, Valencia, Valencian Community, Spain

Dear reviewer, Many thanks for the review and comments. Below, you can find my response to the action items:  

1. *Thanks for the observation; the paper has been updated with two new paragraphs, one for each pending step.*

 

1. *The ontologies are open, and everyone can check them; the visualisation tool (supporting tool) also has a statistics component to display this information; however, the addition of a summary table indicating, for each module, the number of classes and properties is a valuable enhancement. Therefore, a new section has been included (section 3.5) including such information*

 

1. *Discussion about the profile is also included in this new section (3.5) with two new paragraphs. Thanks for highlighting it. Even if the ontologies are not large, we opted for OWL 2 EL profile (as it is commonly used in hierarchical structures) in case it attracts attention and grows.*

 

1. * The current PURL link redirects to a dedicated server at UPV premises that sometimes experiences electricity outages. To avoid these outages, I updated PURL to redirect directly to the GitHub repository containing the TTL files. As for the licenses, ontologies are typically distributed as CC BY 4.0, whereas the supporting tools follow an Apache 2 license. Our main goal is to facilitate the reuse and adoption of both ontologies and supporting tools.*

 

1. *A screenshot and an explanatory paragraph has been added to the paper. The visualisation tool is based on Webvowl. This technology first converts the TTL files into JSON objects and then renders them into visual objects with which users can interact. There is a generic example at* *https://service.tib.eu/webvowl/* *. Adaptation of this original code from GitHub (* *https://github.com/VisualDataWeb/WebVOWL* *) has been performed for the EIFFEL project, and there is a demo version at* *https://eiffo.satrdlab.upv.es/* *(just select ontology from the Menu)*
